# Supplementary material for: A numerical framework for mechano-regulated tendon healing—Simulation of early regeneration of the Achilles tendon
Source: PLoS Comput Biol. 2021 Feb 8;17(2):e1008636. doi: 10.1371/journal.pcbi.1008636 (PMC7901741; doi:10.1371/journal.pcbi.1008636)
Supplement: S1 Table — In vitro strain-stimulation of fibroblasts from various sources. Collagen type 1 and 3 production is expressed as a relative increase to collagen content levels measured without strain stimulation measured after 12–48 hours of strain stimulation. (DOCX) [file pcbi.1008636.s001.docx]

**A numerical framework for mechano-regulated tendon healing – simulation of early regeneration of the Achilles tendon**

Thomas Notermans^1*^, Petri Tanska^2^, Rami K Korhonen^2^, Hanifeh Khayyeri^1^, Hanna Isaksson^1^

^1^Department of Biomedical Engineering, Lund University, Lund, Sweden

^2^Department of Applied Physics, University of Eastern Finland, Kuopio, Finland

^*^Corresponding author

E-mail: [thomas.notermans@bme.lth.se](mailto:thomas.notermans@bme.lth.se)

**S1 Table. Summary of the literature data used for designing the strain-dependent collagen production laws.** In vitro strain-stimulation of fibroblasts from various sources. Collagen type 1 and 3 production is expressed as a relative increase to collagen content levels measured without strain stimulation measured after 12-48 hours of strain stimulation.

| **Ref.** | **Cell source** | **Strain** | **Coll. 1** | **Coll. 3** | **Methods** |
| --- | --- | --- | --- | --- | --- |
| [18] | Fetal rat cardiac fibroblasts | 20% | -12%  100% | - | Hydroxyproline assay |
| [22] | Human dermal | 20% | 8%  7%  26% | - | Hydroxyproline assay |
| [37] | Human periodontal ligaments | 10% | 217% | - | Elisa for type 1 collagen |
| [38] | Neonatal rat cardiac | 5% | 21%  58% | 3%  18% | Western blotting |
| [34] | Rat pulmonary artery | 20% | 214% | 313% | Western blotting |
| [39] | Equine tendon flexor tensor fibroblasts | 9% | 79%  -41%  124%  162% | 322%  -69%  46%  173% | Deuterium-proline, scintillation counting |
| [40] | Epitenon tenocytes/sheath fibroblasts | 8% | -3%  77%  118% | - | Elisa for type 1 collagen |
| [41] | Human tenocytes | 4% | 30%  42% | 14%  22% | Procollagen 1 and 3 antibody fluorescence |
| [42] | Rat tendon fascicles | 5% | 10% | - | Deuterium-proline, scintillation counting |
