# Supplementary figures and images for: A numerical framework for mechano-regulated tendon healing—Simulation of early regeneration of the Achilles tendon
Source: PLoS Comput Biol. 2021 Feb 8;17(2):e1008636. doi: 10.1371/journal.pcbi.1008636 (PMC7901741; doi:10.1371/journal.pcbi.1008636)

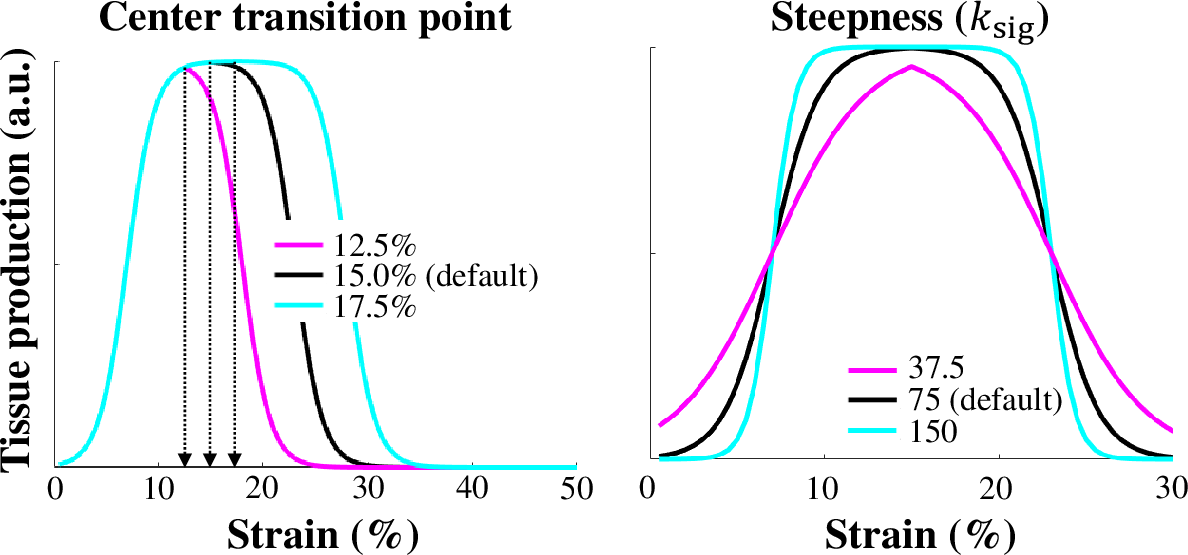

Supplement: S1 Fig — Three different center transition points (12.5%, 15.0% and 17.5%) and steepness (ksig = 37.5, 75, 150) parameters were tested. (TIF) [file pcbi.1008636.s002.tif]

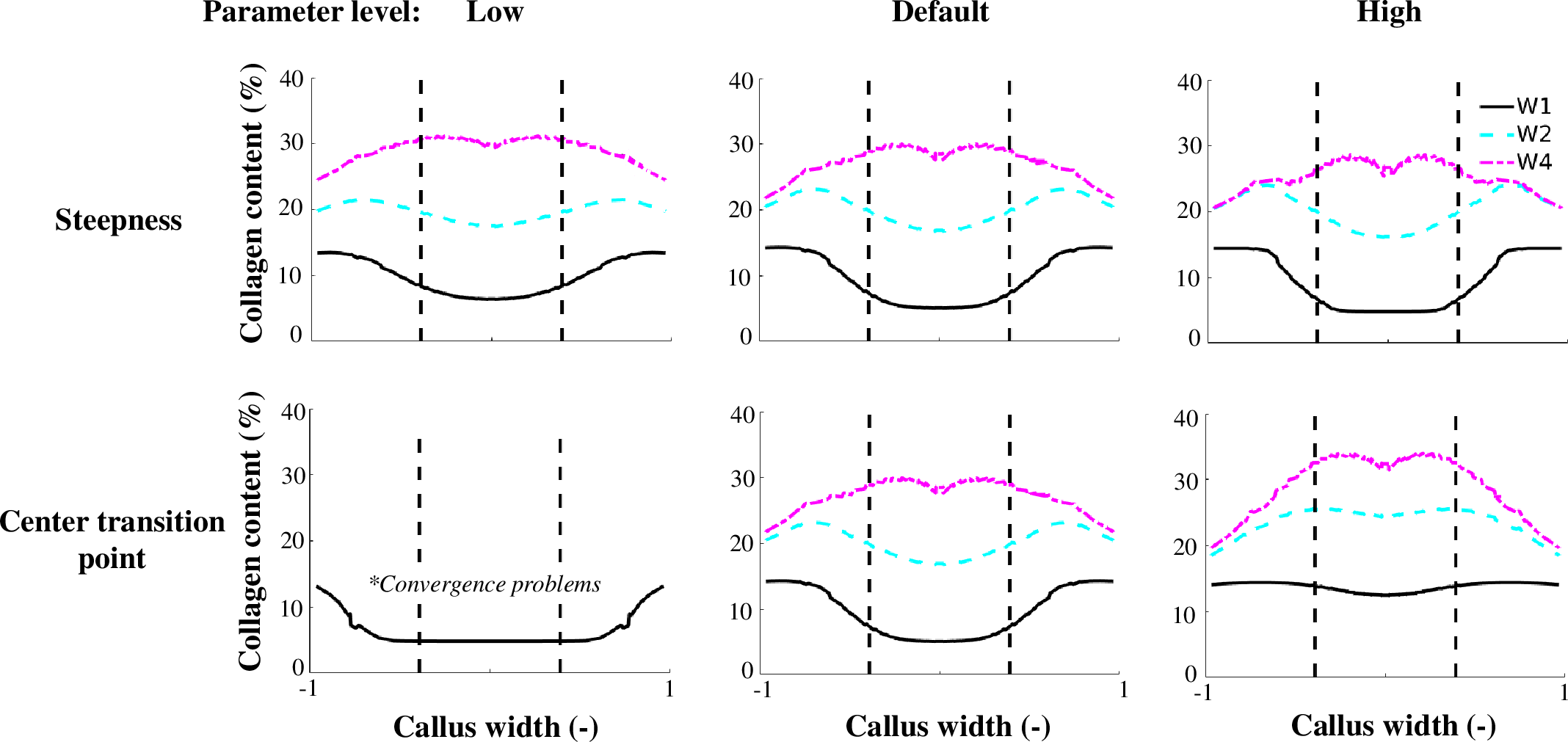

Supplement: S2 Fig — The spatio-temporal evolution of collagen content (% of intact) at week 1, 2 and 4 upon perturbations in the center transition point (low: 12.5%, default: 15.0% and high: 17.5%) and steepness (ksig—low: 37.5, default: 75, high: 150) in production law 2. The width of the stumps is denoted by the black dotted lines. All perturbations of the model parameters predict a decrease in tissue production in the tendon core at week 1. (TIF) [file pcbi.1008636.s003.tif]

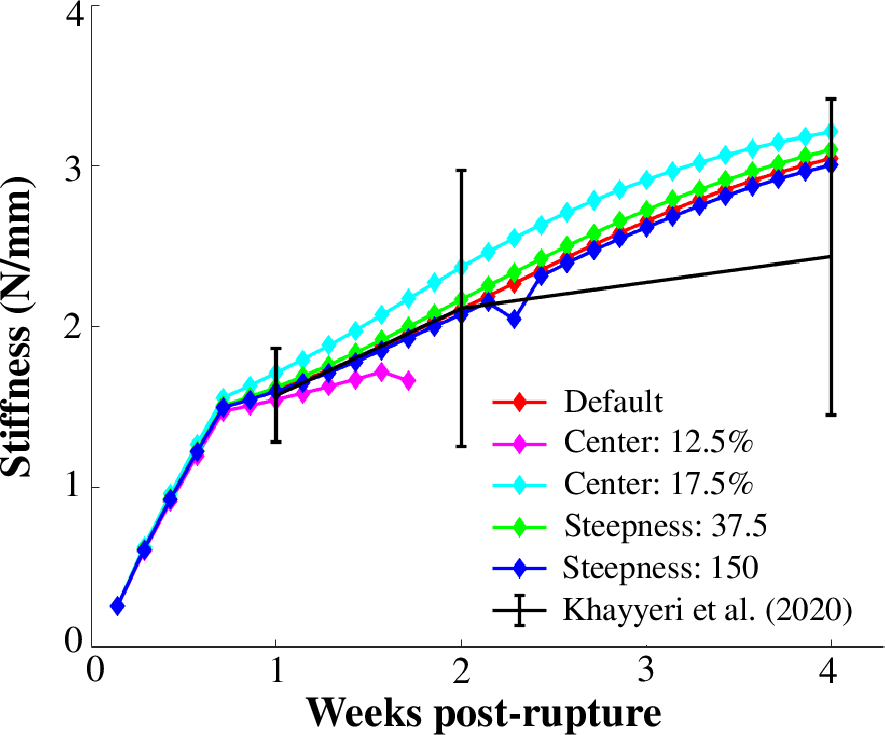

Supplement: S3 Fig — Overall, all different models predicted a development of stiffness in the range of the experimental data. Note that the daily loading simulation for the model with center transition point (12.5%) did not converge beyond 12 days of healing, yet the predicted stiffness was well within the range of experimental data. (TIF) [file pcbi.1008636.s004.tif]

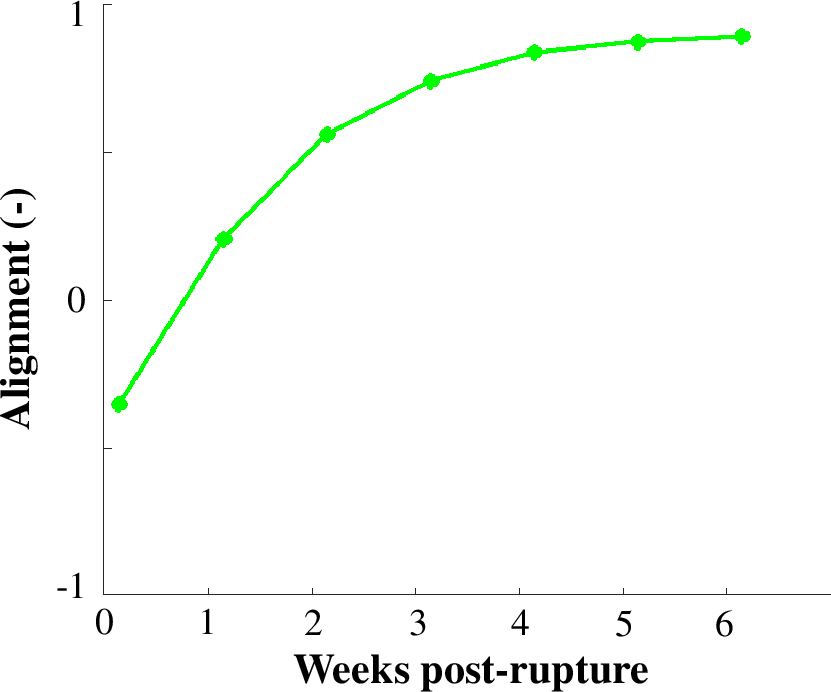

Supplement: S4 Fig — (TIF) [file pcbi.1008636.s005.tif]

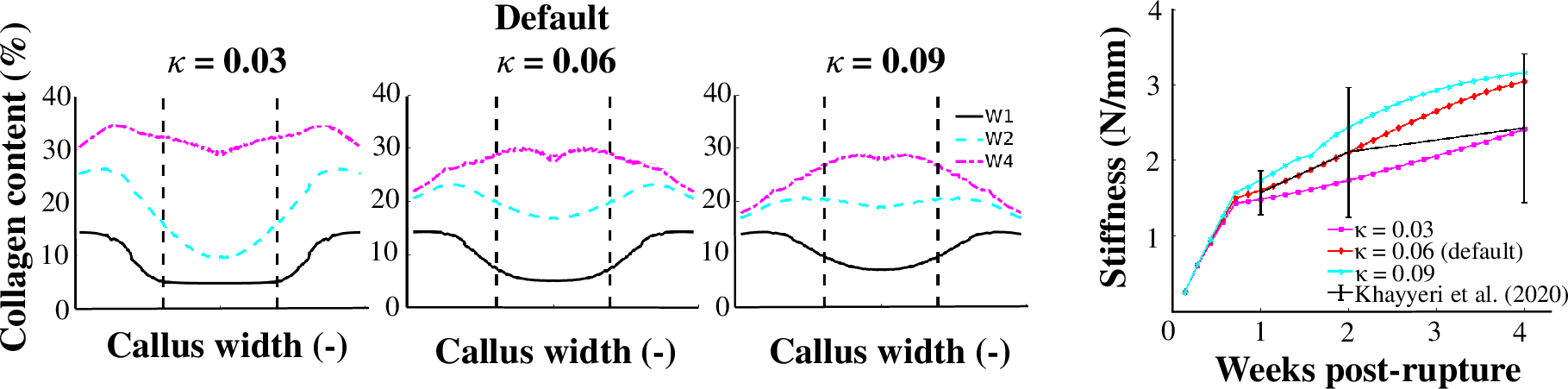

Supplement: S5 Fig — Three reorientation speeds (κ = 0.03–0.06–0.09) were evaluated, such that the majority of reorientation is completed in 6, 4 and 2 weeks, respectively. For each reorientation rate, the evolution of spatio-temporal evolution of collagen content and temporal evolution of stiffness is shown, all with production law 2. The width of the stumps is denoted by the black dotted lines. All models predicted decreased collagen content in the tendon core at week 1 of healing. Additionally, this effect was more prominent and persistent for the slowly reorienting model (κ = 0.03). The stiffness increased with increasing reorientation speed, but the predicted stiffnesses remained within the range of experimental data (Khayyeri et al., 2020). (TIF) [file pcbi.1008636.s006.tif]

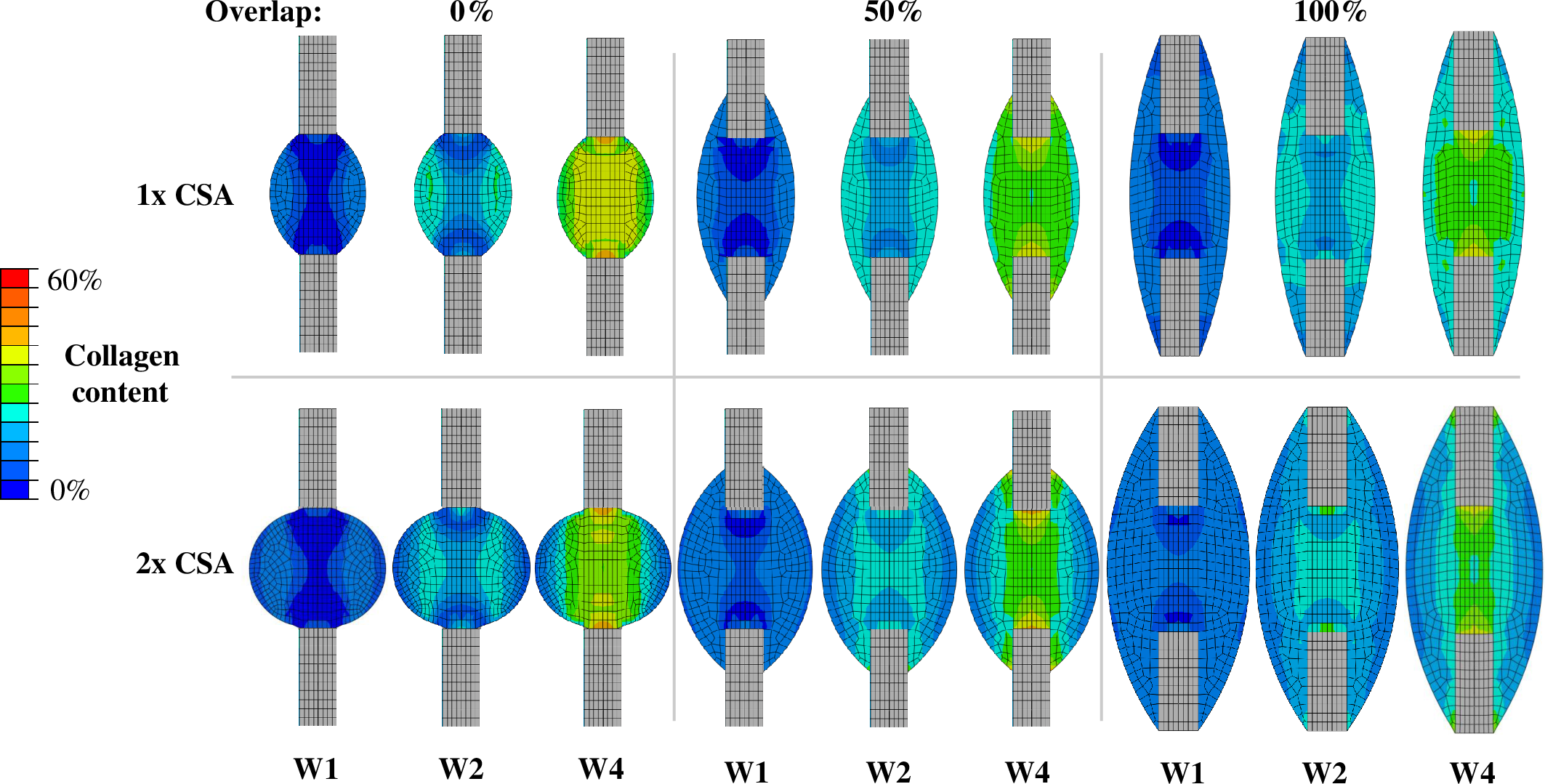

Supplement: S6 Fig — When increasing the stump overlap, the collagen production becomes more homogeneous. (TIF) [file pcbi.1008636.s007.tif]

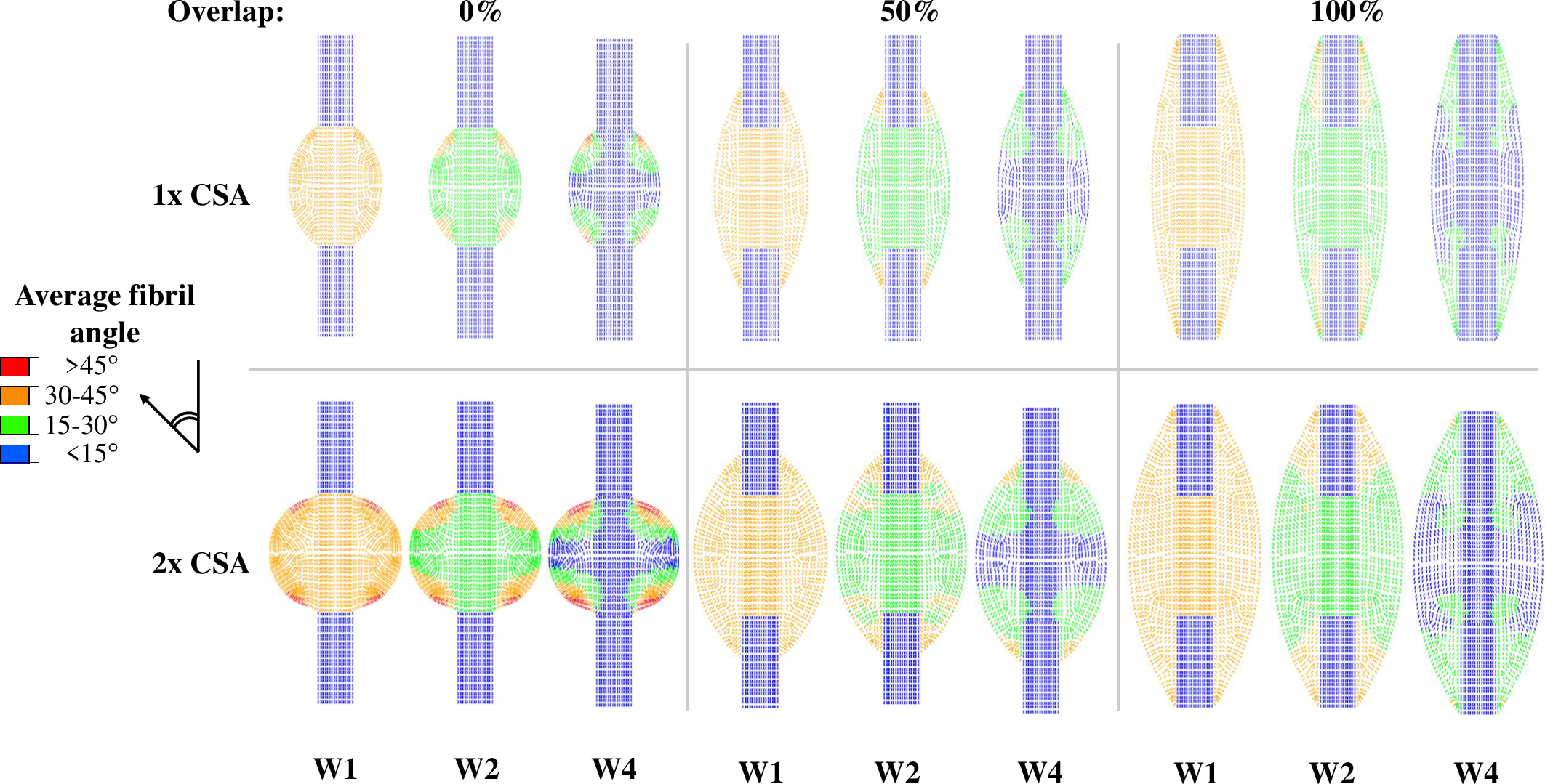

Supplement: S7 Fig — The spatio-temporal patterns of reorientation were similar in all models. (TIF) [file pcbi.1008636.s008.tif]

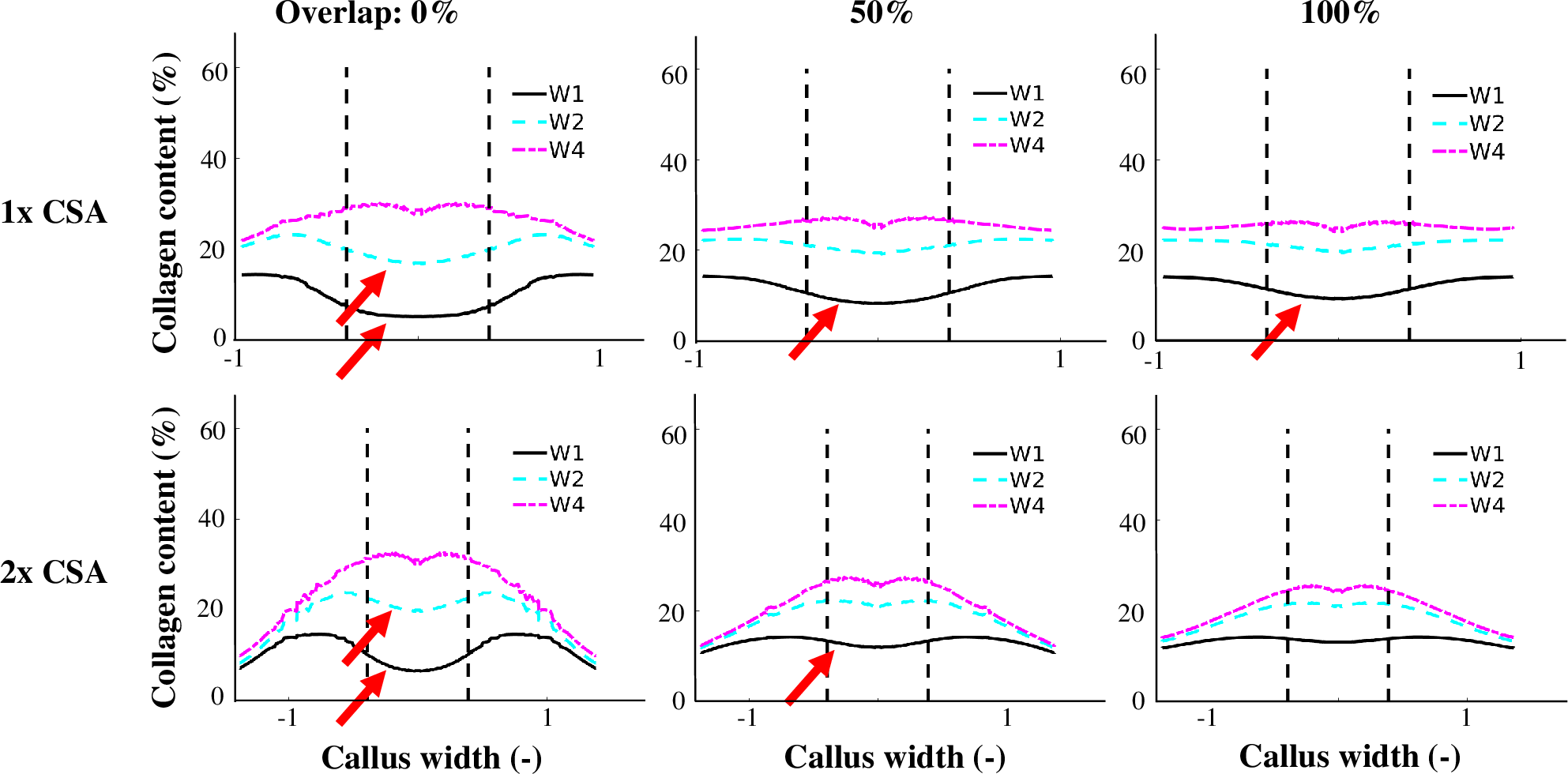

Supplement: S8 Fig — The width of the stumps is denoted by the black dotted lines. Decreased content in the tendon core is denoted by a red arrow. (TIF) [file pcbi.1008636.s009.tif]

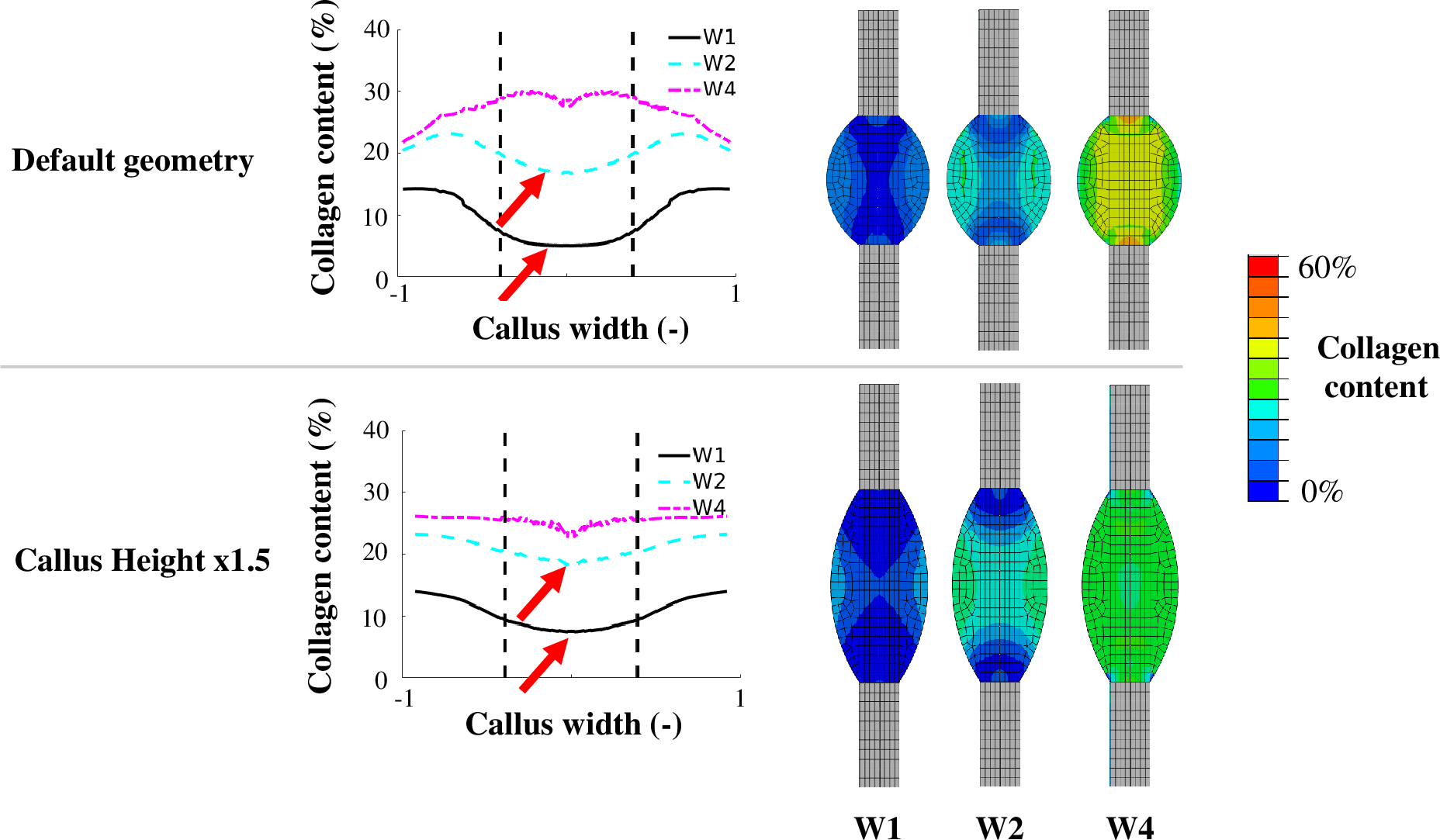

Supplement: S9 Fig — The width of the stumps is denoted by the black dotted lines. Decreased content in the tendon core is denoted by a red arrow. The model with increased callus height predicted a more homogeneous collagen production. (TIF) [file pcbi.1008636.s010.tif]

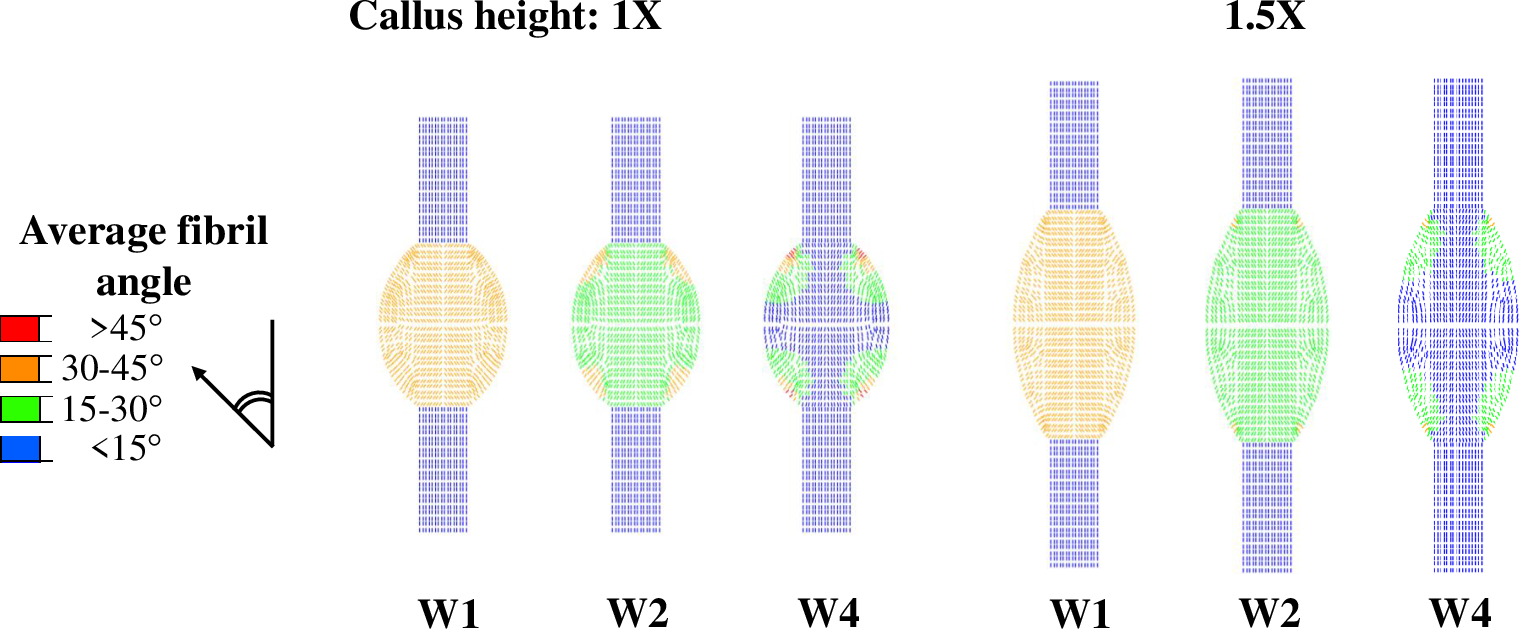

Supplement: S10 Fig — The model with increased callus height displayed a very similar evolution of the spatial distribution of collagen alignment. (TIF) [file pcbi.1008636.s011.tif]

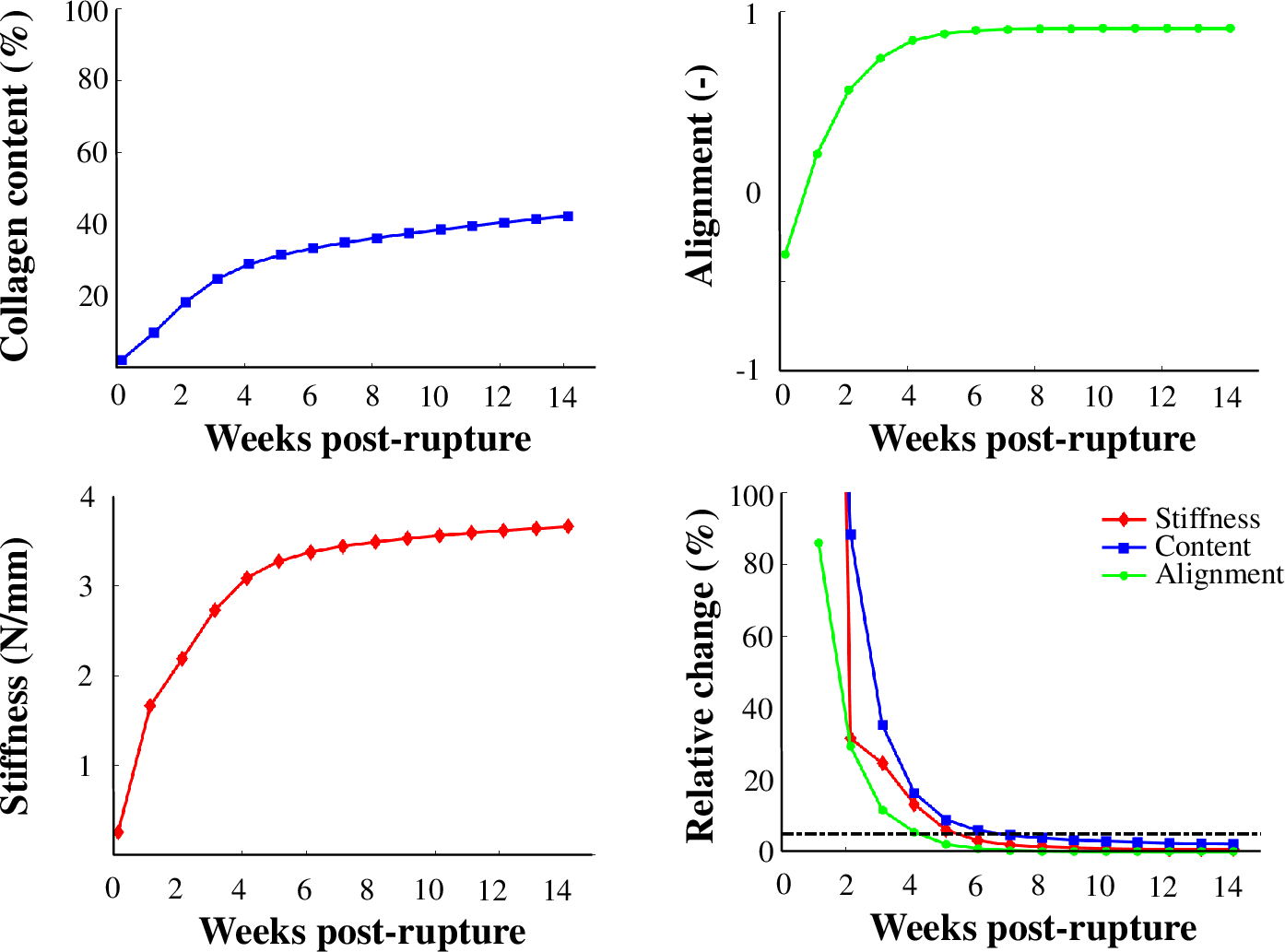

Supplement: S11 Fig — To characterize the long-term predictions of the current healing framework, the model with production law 2 ran for 100 days (~14 weeks). All monitored properties (mean tissue content, alignment, and stiffness) approached steady-state within 7 weeks of healing. The dashed line represents 5% relative change. Within 4 to 7 weeks, all properties changed less than 5% with respect to previous week. (TIF) [file pcbi.1008636.s012.tif]
