# Supplementary material for: A numerical framework for mechano-regulated tendon healing—Simulation of early regeneration of the Achilles tendon
Source: PLoS Comput Biol. 2021 Feb 8;17(2):e1008636. doi: 10.1371/journal.pcbi.1008636 (PMC7901741; doi:10.1371/journal.pcbi.1008636)
Supplement: Attachment — Submitted filename: Rebuttal.docx [file pcbi.1008636.s013.docx]

**Initial title: "A numerical framework for mechano-regulated tendon repair – simulation of early healing of the Achilles tendon"**

**Revised title: “A numerical framework for mechano-regulated tendon healing – simulation of early regeneration of the Achilles tendon”**

Submitted 17^th^ of July; Reviewers’ comments received 24^th^ September 2020: (*Our response in red, and proposed textual changes in blue*)

**Editor**

This manuscript demonstrates novelty and has many merits of interest to the field of tendon biology. The reviewers highlight ways in which the model can be clarified and further justified to improve the quality and potential impact of this work. Namely, including a discussion about the limitations of the model, further justify or revise assumptions, address how temporal changes in tendon stiffness can influence the model, and perform sensitivity analyses related to orientation.

We thank the editor and reviewers for their valuable input and we have tried to carefully respond to all suggestions and remarks, and amended the submission accordingly. We mainly provided additional clarification and discussion in the manuscript and expanded the supplementary material with several sensitivity analyses and more characterization of our modeling framework.

**Reviewer #1:**

In this study, the authors developed a computational modeling framework to investigate the spatio-temporal patterns of collagen synthesis (and the resulting changes in stiffness) in the healing Achilles tendon. According to their simulations, which utilized two different strain-dependent collagen production laws, spatial variations in strain across the healing tendon led to reduced collagen synthesis in the tendon core during early stages of healing. These findings are consistent with experimental observations. In addition, the model predicted the evolution of tendon stiffness after rupture somewhat accurately.

This well-written study provides a possible explanation for the well-documented spatial heterogeneity of healing tendons and highlights the importance of mechanical loading in tendon regeneration. Thus, the findings of this study are of interest to the field. However, some key details about the model are missing, some important sensitivity analyses have not been performed and whether the model reflects a realistic mechanobiological response is not clear.

We thank the Reviewer for the detailed analyses of our model. We have clarified different modeling features in the manuscript, provided additional simulation data in this response document and we have extended the supplementary material with requested sensitivity analyses.

Specifically, the following concerns should be addressed by the authors:

1) According to both production laws used by the authors, the collagen production rate is always greater than (or equal to) zero. Thus, a cyclically loaded tendon never reaches an equilibrium “attractor state” where (net) collagen production ceases. That is, in their model, a cyclically loaded (healing) tendon will continue to produce collagen and stiffen indefinitely. This behavior is clearly not physically realistic. Other models typically allow for reduction of collagen content due to proteolytic activity for certain levels of strain (e.g., when strain levels are below a threshold). In this way, the material properties adapt until equilibration when the optimal strain state is reached. Can the authors comment on this key limitation of their model? Why did they choose to not allow for decreased collagen content under some conditions?

We focused on modeling the initial healing phase, and therefore on tissue production. We agree that for simulating the long term homeostasis, the model would need to include remodeling laws that would also include matrix degradation. However this was not the main focus in this paper. This type of remodeling laws have been investigated e.g. by Richardson et al., (2018). Yet, in our healing model, when strain levels drop below 5%, the resulting production is minimal. Thus, the collagen production and stiffening of the tendon ceases.

To show this, we ran the models with production law 2 up to(14 weeks. All main output parameters (average content, alignment and stiffness) are affected mostly during the first 4-6 weeks of healing and change less than 5% (compared to the previous week) thereafter.

We added this figure as well as the temporal evolution of the mean content, alignment and stiffness to the supplemental material. In addition, we added the following statement to the discussion:

“Predicting long-term homeostasis was not within the scope of this paper. Therefore, the model did not incorporate collagen degeneration (e.g. due to proteolytic activity; Richardson et al., 2018). Still, we identified that the model approaches a steady-state after 6 weeks of healing as the mean content, alignment and the stiffness at that point changed less than 5% compared to the previous week (Fig 1 in S4 Text). However, the model is flexible and we will address remodeling and degeneration in the future.”

2) The authors perform a sensitivity analysis to determine how changes in production law 2 influence collagen content across the callus (Figure S2). However, it would be helpful if they also show how these changes influence the temporal evolution of tendon stiffness. If the production law is altered, are the time-dependent changes in tendon stiffness still consistent with experimental findings?

In addition to supplemental material (Fig 2 in S1 Text), we have now also quantified how perturbations (in center point and steepness) in the mathematical description of the strain-dependent production law 2 affect the temporal evolution of the tendon stiffness. All perturbations in the production law resulted in minor changes and the predicted stiffness values remained within the experimental range. Note that the model (Center: 12.5%) showed numerical convergence issues after 12 days of healing, yet the initial results indicate that the model would behave similarly to all other models, with slightly decreased predicted stiffness values that would still fall within the experimental range at 2 and 4 weeks post-rupture.

This has been clarified in the manuscript as:

”In addition, we investigated the shape of production law 2, as described in Supp S0. However, all perturbations of production law 2 (Fig 1 in S1 Text) predicted decreased collagen production in the callus core during early tendon healing (Fig 2 in S1 Text) and only minor changes in the temporal evolution of stiffness (Fig 3 in S1 Text).”

We also added this sensitivity analysis to supplemental material as Fig 3 in S1 Text.

3) It is described that the reorientation rate was chosen to match findings in the literature. But there is no sensitivity analysis performed to determine how changes in reorientation rate influence key output parameters.

We performed a sensitivity analysis on the rate of reorientation for production law 2. The main trends observed in the collagen content remain similar and the temporal evolution of stiffness remained within the experimental range.

We added the following statement in the discussion: “Sensitivity analysis of the rate of reorientation showed only slight changes in the observed trends for the spatio-temporal evolution of the collagen content and temporal evolution of the stiffness (Fig 2 in S2 Text).”

We also added this sensitivity analysis to supplemental material (Fig 2 in S2 Text).

4) It appears that the model includes two forms of adaptation: strain-regulated collagen synthesis and collagen reorientation that in turn alters the mechanical response of the healing tendon through the fiber-reinforced model. It would be helpful and interesting if the authors delineate the importance of each of these forms of adaptation by performing simulations (a) with only strain-regulated collagen synthesis and (b) with only collagen reorientation. Does the model’s agreement with experimental data depend on “turning on” both of these forms of adaptation?

To investigate the effects of the production and reorientation separately we ran the models with production law 2 with a) *only production*, b) *only reorientation*.

a) The result from the *only production* case displays that incorporating the collagen production alone in the model would predict production only in the callus periphery. This is not enough to increase the overall effective stiffness of the callus to decrease the strains in the tendon core to reach strain levels where collagen production is predicted (<20%). This result shows that collagen reorientation/alignment plays a substantial role in increasing the effective stiffness of the tissue in the direction of loading. Subsequently, upon the reorientation of collagen fibrils, the increase of the collagen content would also increasingly affect the tissue stiffness. This clearly shows that we cannot capture the experimental data found in Khayyeri et al. (2020; spatio-temporal content evolution and temporal evolution of stiffness) considering only production without reorientation.

b) The result from the only reorientation model case (without production) resulted in a callus where the collagen content remained at 1% density, which leads to convergence problems upon external loading >0.5N.

As a *proof-of-concept* we also ran the *only production*, default, and *only reorientation* models, with an initial callus density of 10% (instead of the default 1%). These modeling results showed that the incorporation of both production and reorientation increase the stiffness of the tendon in a nonlinear fashion. Reorientation also affects the spatial effects on collagen content that are more visibly seen in later time points of healing.

5) In many of the studies cited to justify the strain-dependence of collagen production, strains were directly applied to cells on a flexible substrate. In healing tendons (as in other fibrous tissues), tissue- and matrix-scale strains are not equal to strains experienced by resident cells. Instead, tissue- and matrix-scale strains are often amplified or attenuated across length scales (see, for example, PMID 26726994). Thus, it may not be appropriate to rationalize tissue strain-dependent collagen production in healing tendons based on data from directly deformed cells.

The reviewer raises a good point. We based the strain-dependent collagen production laws indeed on *in vitro* experimental data, as is custom in this type of modeling (see e.g. Richardson et al., 2018; Chen et al., 2018). These *in vitro* experiments with cells on flexible substrates have also been shown to display quite significant strain transfer. For example, Wall et al. (2007) found an average strain transfer of 52-74% between the local substrate strain and cellular strain. The reference provided by the reviewer (PMID 26726994) found strain transfer of 60-70% (matrix vs. cell strain) and ~40% (applied vs. cell strain) in bovine outer meniscus specimens. Screen et al. (2003) found that cell-level strains were about 25-50% the applied strain in rat tail tendon and bovine tendon. In another numerical study, Achilles tendon healing was implemented using a constant strain transfer of 28% between applied and cell-level strains (Chen et al., 2018).

Since a thorough characterization of strain transfer across multiple length scales is lacking, and the available data implies similar strain attenuation in vitro and in vivo experiments we did not implement an additional strain transfer feature. Additionally, available *in vitro* data (Wall et al., 2007) displayed 52-74% strain transfer (substrate vs. cell strain) which is of a similar magnitude as found between local matrix strain and cell strain (60-70%) found in (PMID 26726994). Thus we assumed that the strain transfer in our production law in the (based on *in vitro* work) was similar to the strain transfer from local tissue strain (calculated in our finite element models) to the cellular strain.

Additionally there is a lack of experimental data on strain-dependent collagen production in 3D tendon-like tissues. We are aware that this data is important and we are therefore trying to establish this experimental work to obtain this data to verify the trends we observe in the previous *in vitro* work that we currently used to design our production laws.

We added the following statement to the discussion: “Different levels of strain transfer have been observed in soft tissues, e.g. tendon (Screen et al., 2003) and meniscus (Han et al., 2016). Similar levels of strain transfer have been identified in in vitro experiments that apply strains to cells on a flexible substrate (Wall et al., 2007). Another numerical Achilles tendon healing study implemented a constant strain transfer of 28% between tissue and cell-level strains (Chen et al., 2018). However, since a thorough characterization of strain transfer across multiple length scales is lacking, and the available data implies similar strain attenuation in *in vitro* and *in vivo* experiments, we did not implement strain transfer from the matrix-level to the cell-level strains.”

6) Was a convergence analysis performed to ensure that the mesh was sufficiently dense?

Yes, a mesh convergence analysis was performed. Below, we display the strain field for a variety of mesh densities. The strain field is similar for all meshes with higher densities than our default mesh.

We added the following statement in the methods section: “To explore the effects of our modeling assumptions we performed sensitivity analysis on the mesh density, mesh geometry, loading rate, reorientation rate, exact definition of production law 2, and long-term predictions.”

In addition we added a remark in the results section: “A mesh convergence study showed that an increasing mesh density was not found to significantly affect the accuracy of the predicted strain maps. ”

7) The authors should include the constitutive equation that they used in the main manuscript rather than simply providing a reference.

According to the Reviewer’s suggestion, we added the main constitutive equations of our material model in the methods:

“Briefly, the material behavior of the collagen fibrils was modeled according to the viscoelastic standard linear solid (SLS) model:

$P_{s}=\left\{ \begin{matrix} E_{s} \left( e^{K_{s}\varepsilon_{s}}-1 \right), \varepsilon_{s}>0 \\ 0, \varepsilon_{s}\leq0 \end{matrix} \right. (1)$

$$P_{\eta}=\text{η}\frac{d\varepsilon_{\eta}}{dt}, \left( 2 \right)$$

$P_{f}=P_{s}+P_{\eta}=P_{s}+ \text{η}\frac{d\varepsilon_{\eta}}{dt}, \left( 3 \right)$

where $P_{s}$, $P_{\eta}$and $P_{f}$are the first Piola-Kirchhoff stress in the springs, dashpot and the whole SLS model, respectively, $E_{s}$ and $K_{s}$ are the linear and exponential modulus parameters, $\text{η}$ is the viscosity of the dashpot, $\varepsilon_{s}$ is the strain in the spring, and $\varepsilon_{\eta}$ is the strain in the dashpot. Subsequently, the Cauchy stress per collagen fibril is defined according:

$$\boldsymbol{\sigma}_{f}={\frac{\lambda}{J}P_{f}\vec{\boldsymbol{e}_{f}} \vec{\boldsymbol{e}_{f}}}^{T}\mathbf{,}\left( 4 \right)$$

where $\lambda=\frac{\left\| \vec{\boldsymbol{e}_{f}} \right\|}{\left\| \vec{\boldsymbol{e}_{f,0}} \right\|}$ is the fibril stretch, $\vec{\boldsymbol{e}_{f}}$ and $\vec{\boldsymbol{e}_{f,0}}$ are unit vectors describing the current and initial fibril direction respectively. The non-fibrillar ground substance was modeled by an orthotropic strain-energy formulation:

$W_{ort}=\frac{1}{2}\sum_{i,j}^{3} a_{ij}\mathrm{tr}(\boldsymbol{E}\cdot\boldsymbol{L}_{ii})\mathrm{tr}(\boldsymbol{E}\cdot\boldsymbol{L}_{jj})+\sum_{i,j\neq1}^{3} G_{ij}\mathrm{tr}(\boldsymbol{E}\cdot\boldsymbol{L}_{ii}\cdot\boldsymbol{E}\cdot\boldsymbol{L}_{jj}) ,$	(3)

that relates the stiffness parameters ($a_{ij}$, $G_{ij}$) to the Green-Lagrange strain tensor ($\boldsymbol{E)}$using an orthogonal base formulation where $\boldsymbol{L}_{ii}$ represents a dyadic product of a set of orthogonal unit base vectors. This material definition allows the ground substance to have different material properties (modulus and Poisson's ratio) in the longitudinal (2-direction) and transverse plane (1- and 3- direction). The Cauchy stress for the non-fibrillar ground substance was calculated according:

$$\boldsymbol{\sigma}_{m}=\frac{1}{J}\boldsymbol{F}\cdot\frac{\partial W}{\partial\boldsymbol{E}}\cdot\boldsymbol{F}^{T}, (5)$$

with deformation gradient ($\boldsymbol{F}$) and jacobian ($J=det(\boldsymbol{F})$). We modeled strain-dependent fluid flow using a poroelastic formulation following Darcy’s law with a void ratio dependent permeability of the tendon (additional details in [26]).”

**Reviewer #2**

This interesting study proposes a mechano-regulatory framework to describe spatio-temporal evolution of collagen content and alignment and the temporal evolution of stiffness during tendon healing. The novel methodology includes strain-regulated laws to compare tendon callus properties in the core and peripheral regions. Collagen production in the tendon core was delayed and decreased throughout the first 2 weeks following tendon injury. Favorable comparisons of the model outputs to experimental data indicate preliminary reliability of this model.

The novelty of this work regards the spatial and temporal descriptions of callus deformation and the consideration of supraphysiologic levels of strain. While it is fully understood that the reliability of a numerical simulation is gauged from comparison of its predictions to available experimental results, it is felt that this model provides only a modest advancement. Specifically, the model considers only the callus, with little detail paid to the boundary conditions (e.g., adjoining tendon stumps) aside from the callus-stump interfaces. The model also relies on a large number of assumptions (e.g., apparently no distinction between the fibrillar collagens) and the loading conditions (lines 150-153) are not well justified.

We thank the Reviewer for the critical comments on our work. We believe that the heterogeneity of tendon healing properties is underappreciated in earlier available experimental work. However, we recently performed one of the first experimental studies that quantify this heterogeneity of collagen during early tendon healing. In alignment with this novel experimental data, we now present the first tendon healing model that explores the 3D heterogeneity and thereby both spatial and temporal evolution of important tendon properties (collagen content, collagen alignment and overall biomechanical properties).

Indeed, we acknowledge that factors such as the callus stumps, different fibrillar collagen types, other biological stimuli (blood/oxygen supply), damage, tissue differentiation, may play an important role in tendon healing. Yet, in modeling, a key principle is Occam’s razor, which means that models should be kept as simple as possible to explain most effects with the least amount of parameters as it is more likely that a simple explanation is the right one. Moreover, developing a model with too many complex parameters at once makes it very challenging to assess the importance of each feature. Unfortunately, there is also a lack of adequate experimental data to address these additional features mentioned. Therefore, accounting for these additional processes would be too speculative at this point since our understanding of underlying mechanisms is still too limited.

We have tried to justify and clarify our modeling choices in the manuscript upon the reviewer’s valuable comments. Most importantly, computational modeling efforts can aid in highlighting key knowledge gaps for the development of experimental and numerical work. Additionally, upon validation of some important output parameters, these modeling efforts can be utilized to test a wide range of possible experimental scenarios that could never be tested in an actual experimental set up due to limited resources. In this light, this work could for example be utilized in the future to identify possible optimal mechanical loading regimens during healing that may stimulate tendon healing, which could be verified by experimental work. Similar approaches have been utilized by cartilage, bone and cardiovascular research field to help understanding the evolution of disease, understand important processes of tissue regeneration, and ultimately improve clinical treatments.

In this broader perspective, this study provides a framework that can investigate tissue differentiation, tendon (re)rupture and help in identifying optimal rehabilitation regimens. In the future, we hope that our experimental work and the work of other research groups will provide more information on these other processes mentioned by the reviewer, such that we can elucidate and implement their role in our computational framework. The authors believe that the introduction and development of this framework is essential to push scientific advancement in the field of tendon mechanobiology. We have considered all comments of the reviewer and we elaborate in the following section.

Specific Comments:

ABSTRACT
Lines 35 -38: restate this sentence to convey that your results (biomechanical and collagen orientation) suggest improved healing

We want to clarify that our modeling results do not suggest improved healing. However, we have rather modeled different mechanisms in healing and captured the main trends observed in collagen alignment and production as observed in experimental data.

INTRODUCTION
General comment:
By “tendon repair,” are the authors alluding to full width transection models with or without surgical repair?

By tendon repair, we mean the innate healing process after full-width transection without surgical repair. For clarification, we have adapted the manuscript and used the term “tendon healing” instead.

We added the following statement in the introduction: “In particular, we modeled the healing of a full-width transection-like rupture without surgical repair.”

Lines 71-72: for reference 11, is the structural analysis based on collagen fibers or fibrils, or both?

The structural analysis in Khayyeri et al. (2020) entails characterization of the collagen fibril scale by small-angle X-ray scattering (SAXS). This scattering method is sensitive to the periodic D-spacing caused by staggered organization of collagen molecules making up collagen fibrils. In addition, SAXS can be utilized to quantify fibril alignment, fibre delamination, interfibrillar ordering, anisotropy.

We amended the text to clarify: “Different structural collagen properties in healing transected rat Achilles tendon, measured e.g. on the fibril-scale by Small-Angle X-ray Scattering (SAXS) and on the tissue-scale by histology, implied decreased collagen levels and delayed maturation in the center of the tendon callus (core) during the first 4 weeks of healing (Fig 1) [11].”

More information on this technique from our group:

Khayyeri et al., 2017, Achilles tendon compositional and structural properties are altered after unloading by botox. *Scientific reports* 7:13067

Turunen et al., 2017, Effects of tissue fixation and dehydration on tendon collagen nanostructure. *Journal of structural biology* 199:209-215

Line 72: consider removing “see” from (see Fig 1)

We adapted the manuscript according to the reviewer’s comment.

Lines 78-79: consider replacing “histology work” with “histologic analysis.” What type of collagen (I, III) staining are you referring to?

We changed our formulation according to the Reviewer’s comment. In Best et al. (2020), a Masson’s Trichrome staining was performed to characterize collagen content and organization (see figure below). To our knowledge, differentiation between collagen type-1 or 3 is not possible with this staining.

Line 89: remove “Only” from “only few”

We adapted the manuscript according to the reviewer’s comment.

Line 104: it is assumed that collagen production (which of course is a central focus of the current study) refers to biochemical compositional measures of collagen and not collagen gene expression. Furthermore, is it an accurate statement that the authors are not distinguishing collagen I from collagen III (or other fibrillar collagens)? Col III matrix tensile properties differ from those of Col I. Is it necessary for the model to account for the gradual temporal reduction in Collagen type III:I ratio that presumably occurs during the healing process? Please clarify these points.

The reviewer raises good points. We agree that the different roles of collagen type-1 and 3 are of importance in tendon healing. Initially, we intended to investigate and model collagen 1 and 3 separately, however, after a thorough literature study on the matter many questions remained unanswered:

- There is no elaborate quantitative or qualitative characterization of the spatio-temporal distribution during tendon healing for collagen type-1 and 3. However, we are currently addressing this and animal experiments to characterize this are ongoing.
- The difference in the mechanical properties of the different collagen types is not thoroughly characterized.
- It is not clear how mechanical stimuli (i.e. strain) and/or inflammation affect the production of collagen types 1 and 3.
- Is collagen type-3 removed/degraded or integrated into collagen type-1 fibres?

Most probably, collagen type-3 is produced in the early healing phase in a random or isotropic manner to establish an initial stable matrix network that allows cellular processes such as *migration* and *mechanosensing*. We capture this initial behavior in our model by creating an initial network to be random/isotropic, with decreased mechanical properties (more collagen type-3 like) that develops into an aligned and stiff matrix (more collagen type-1 like).

We added the following text in the discussion: “It is also known that distinct collagen types (type 1 and 3) are observed during tendon healing. Yet, we currently only considered the total collagen phase (not accounting for the different collagen types) to capture bulk tissue production during healing. This was because important information about the different collagen types during tendon healing are currently not known, e.g. the spatio-temporal distribution of the different collagen types, the mechanical properties of the different collagen types, and the potential differences in the collagen production (and degeneration) mechanisms. Therefore, we assumed only a single type of collagen to capture bulk tissue production during healing. However, the different roles and properties of collagen type-1 and 3 will be investigated in future studies.”

Line 135: please provide a rationale for the prescribed zero pore pressure.

From poroelastic theory, a zero pore pressure is prescribed to the external surface of objects to ensure a so-called *drained* state, which allows fluid to flow in and out of the body, and thus also allowing volumetric changes in the material. We previously used this zero pore pressure boundary condition to mimic the experimental test set-up when the tendon is mechanically tested without any further surrounding tissue where the fluid flow can easily occur (Khayyeri et al., 2015; Khayyeri et al., 2016; Notermans et al., 2019).

To clarify this, we added the following sentence in the caption for Figure 3: “and zero pore pressure is prescribed to the nodes on the outer surface to allow free fluid flow.”

To characterize the effect of the prescribed pore pressure, we explored various magnitudes of external pore pressure and observed that it did not affect the predicted strain field. Below, we ran the first iteration of the model with production law 2 with changes in the boundary condition value for the pore pressure. This had no noticeable effect on the pore pressure and strain field at the end of loading, this may be due to minimal volume change.

Lines 145-146: is sigma in these equations a generic variable for tendon properties (many readers may associate sigma with mechanical stress)

We would like to emphasize that indeed, sigma is associated with mechanical stress. We have clarified this in the manuscript in the methods section: “For the healing callus, a density function 𝜌 was introduced to scale the Cauchy stress exerted by the collagen and ground substance in intact tendon to callus properties according:”.

Lines 147-9: please clarify this approach. It is unclear (to this reviewer) how a callus density of 1% of the intact tendon matches the d3 tendon stiffness. The cross-sectional area presumably is also altered (and modeled) from experimental data? What is the normalized stiffness at d3 post-injury? Please provide a justification for the selection of 13 random initial fiber directions.

The normalized stiffness was defined as the stiffness measured in the healing tendon normalized by the stiffness of intact tendon. Eliasson et al. (2009) reported a stiffness at day 3 post-rupture that was ~5% of intact tendon stiffness. Subsequently, we calibrated the callus density to match this finding by Eliasson et al. (2009) to get an approximation of the magnitude of the post-rupture stiffness of the tendon at the earliest timepoint (3 days), whereas the stiffness in Khayyeri et al. (2020) was measured earliest at 1 week.

We amended the text in the discussion accordingly: “However, we calibrated the initial callus density (1%) such that the initial stiffness (5% of intact tendon stiffness) after transection at day 1 was based on the earliest available mechanical data [6].”

We created the model geometry, e.g. cross-sectional area and height of the callus, according to the animal experiments in Khayyeri et al. (2020), to which our simulation results were compared to. Yet, our model geometry did not change over the coarse of the healing process, whereas the experimental data showed temporal changes in both cross-sectional area and height of the callus. Future modeling efforts may include temporal adaptation of the geometry of the callus, however, as an initial assumption we kept the geometry constant throughout the whole healing process and instead quantified how different geometries may affect healing (see S3 Text).

The fibrillar matrix in our constitutive model is based on the approach by Wilson et al. (2004) (also used e.g. in Tanska et al., 2018) for modeling articular cartilage. Those studies showed that 13 fibrils were reasonable to resemble an initially random collagen network matrix accurately (see figure below where blue arrows represent the implemented 13 fibrils in a single material point, and the red arrows represent their mirror illustrating the full symmetry of the fibril directions).

The aforementioned references (Wilson et al., 2004; Tanska et al., 2018) were added to the manuscript as justification for the 13 fibrils.

Lines 150-53: how sensitive is the model output to these tendon loading rates? Does the model only consider the peak daily load (as opposed to the # of loading cycles) which is the mechanical stimulus for collagen remodeling/production?

Regarding loading rate: We have implemented a loading rate that is in the physiological range of tendon loading. However, we explored the model with production law 2 with 3 different loading rates and observed no changes in the trends for the spatio-temporal collagen content predictions and temporal stiffness evolution (see figure below).

Regarding peak load: Currently, state-of-the-art computational adaptive models for simulating remodeling or tissue regeneration utilize the maximum mechanical stimulus as the main parameter governing tissue adaptation. In addition, it is largely unknown how the # of load cycles affect tendon adaptation and to the authors’ best knowledge there is no computational framework that utilizes the # of load cycles to govern tissue production or remodeling.

We added the following statement in the methods section: “Peak daily loading (maximum principal strain magnitudes) guided the heterogeneous mechano-regulated production of collagen and ECM.”

Adaptive mechanobiological model: the authors detail the callus matrix mechanical behavior, but the boundary conditions are minimally addressed. What initial stiffness values are assigned to the adjoining stumps, and do these change over time (post-injury)? Do the intact tendon stumps strain-shield the callus? At strain levels exceeding 15%, it can be argued that collagen fibers may undergo plastic deformation or damage, thereby altering their orientation and load-bearing capacity. Does the model account for the latter occurrences?

These implemented boundary conditions are similar to Notermans et al. (2019) (see below). The tendon stumps are assumed to have intact tendon properties that were determined in Notermans et al. (2019) by using the mechanical data from Khayyeri et al. (2017) and these properties do not change throughout the healing process.

We amended the caption to figure 3 accordingly: “**Fig 3. Geometry, boundary conditions and mesh.** Longitudinal cross-section of the implemented geometry describing the dimensions (mm) of the intact stumps and healing callus (A) and the boundary conditions (B), where a force *F* is applied to the top-surface, the top surface nodes were only allowed to move in longitudinal direction, the bottom surface nodes are clamped, and a zero pore pressure *p* is prescribed to the nodes on the outer surface to allow free fluid flow. The implemented finite element mesh was a quarter of a cylinder, consisting of two intact stumps (denoted in blue) and the healing callus (denoted in red). Using symmetry conditions, this mimicked a complete 3D cylinder.”

The intact tendon stumps may provide strain-shielding if the callus overlaps the tendon stumps (see figure below).

Here we ran the first iteration for the healing model with and without the callus overlapping the intact tendon stumps. For the model with overlap, it is visible that the intact tendon stumps can strain-shield the callus strains for the callus part that protrudes the intact tendon stumps.

Indeed, one of the main results in this study is the extreme strain levels observed during early healing (see figure above), and we can only assume that plastic deformation and damage may play a role there. However, these considerations add another level of complexity to the modeling that is not part of this framework development.

In general, there is a need for experimental characterization of the spatial distribution of strain magnitudes during tendon healing. These data are vital to validate the predicted strain maps but these studies could also provide additional information on the spatial distribution of damage or strain-shielding. However, there is some experimental data available on the maximum strain levels during tendon healing upon mechanical testing. Experimental data for load-to-failure testing implied high tissue level strains (>30%) when comparing clamp displacement to the total tendon height, at 3-21 days post-rupture (32-50%; Eliasson et al., 2009). Experimental data for creep testing at 1 week post-rupture implied high strain levels (~40%) comparing the displacement (at 5N load) to the callus height (Khayyeri et al., 2020). It is reasonable to assume that these strains are heterogeneously distributed which implies that (parts of the) healing callus may experience extreme strain levels and that local plastic deformation or damage in these regions is likely.

Lines 199-200: please provide references for the “well established alignment criterion”

Following the reviewer’s suggestion we added appropriate references in the manuscript:

Hsu H-J, Lee C-F, Locke A, Vanderzyl SQ, Kaunas R. Stretch-Induced Stress Fiber Remodeling and

the Activations of JNK and ERK Depend on Mechanical Strain Rate, but Not FAK. Vooijs M, editor.

PLoS One. Public Library of Science; 2010; 5: e12470.

Jungbauer S, Gao H, Spatz JP, Kemkemer R. Two Characteristic Regimes in Frequency-Dependent

Dynamic Reorientation of Fibroblasts on Cyclically Stretched Substrates. Biophys J. 2008; 95: 3470–3478.

Chen K, Hu X, Blemker SS, Holmes JW (2018) Multiscale computational model of Achilles tendon wound healing: Untangling the effects of repair and loading. PLoS Comput Biol 14(12):e1006652.

RESULTS
Can the model predict callus rupture strain, load, and location? I would expect that most of these experimental data are also available from prior studies. If prior studies reported optical strain during tensile loading, it would be interesting to know whether the core vs peripheral regions exhibited differential strain responses at the different time points.

This model can provide information on rupture strain, load and location upon the derivation of a valid failure criterion, and we believe it would be an interesting future study. However, as of today, there are no spatial measurements for local tendon strain during post-transection rat Achilles tendon healing available. This is one of the main gaps in the current experimental literature that we would like to address in the future. Such studies would improve the possibilities to corroborate and develop our computational model. Instead the focus in the current study is on predicting the adaptation of the tendon properties over time during healing.

Figure 6: please clarify that the temporal nature of collagen content changes for law 1 was limited to the core region (as Figure C does not include a ‘2’ for law 1)

Both production laws predict strong production from the callus stump interface, however, in production law 2 this is preceded by the strong initial collagen production in the tendon periphery.

We clarified the results section according to the reviewer’s comment: “Production law 1 predicts most collagen production in the tendon core at all times.”

DISCUSSION
This reviewer understands and appreciates the authors’ assumption that healing is a strain-driven process (strain-dependent production laws), but I think that the complex interrelationships between collagen content, fiber orientation, stiffness and principal strain warrants additional discussion.
For example, please consider that collagen production and orientation could be influencing the strain and stiffness behavior.

Simply said, increasing collagen production and longitudinal collagen alignment, the overall tendon stiffness increases and thus strain levels decrease. As strain levels decrease, tissue production will decrease. Our model has simplified the complex interrelationship between strain-dependent collagen production alignment, and the resulting tissue stiffness. Yet, a recent study has summarized many strain-regulated mechanisms for regulating collagen content (through production, degradation and damage) and alignment that may play a key role in tendon healing (Richardson et al., 2018).

We utilized our material model (that was previously calibrated to intact tendon properties) to link the collagen content to local stiffness scaling the stress-strain behaviour of the healing callus with the local collagen content (expressed as % of intact content) as discussed in the methods: “Intact constitutive tendon properties were used for the intact tendon stumps [27, 28]. For the healing callus, a density function 𝜌 was introduced to scale the Cauchy stress exerted by the collagen and ground substance in intact tendon to callus properties according:

$\sigma_{\mathrm{callus}}^{\mathrm{collagen}}=\rho*\sigma_{\mathrm{intact}}^{\mathrm{collagen}}$, (6)

$\sigma_{\mathrm{callus}}^{ground substance}=\rho*\sigma_{\mathrm{intact}}^{\mathrm{ground} \mathrm{substance}}$ . (7)”

The material model also considers the different collagen orientations, such that collagen fibrils that are oriented in the major loading direction, take up most load, whereas fibrils that are oriented perpendicular to the major loading direction barely take up any load. Therefore in the initial random configuration there are many collagen fibrils that do not contribute to the stiffness of the material whereas these fibrils contribute more to the effective stiffness of the overall tendon once they align in the direction of maximum principal strain.

We have added the following statement to the discussion in the manuscript: “Every iteration (representing a day of healing), a mechanical load was applied to the tendon to determine the spatial distribution of maximum principal strain magnitude and direction. On a daily basis, the maximum principal strain magnitude and direction govern collagen production and reorientation, respectively. With increasing collagen production and longitudinal collagen alignment, the overall tendon stiffness increases and thus strain levels decrease. As strain levels decrease, tissue production will decrease.”

Lines 320-321: If my understanding is correct, please state more clearly that the non-collagenous tissue composition is not considered in this model.

We would like to emphasize that our material model describes the mechanical contributions of 3 components: collagen matrix, ground substance and fluid. So, we do include non-collagenous tissue composition.

We have clarified this in the method section: “We assumed that the ground substance, i.e. non-collagenous matrix, is produced at a similar rate as collagen, thus the spatio-temporal evolution of collagen and ground substance occur in a similar fashion.”

**Reference list**

Hsu, H. J., Lee, C. F., Locke, A., Vanderzyl, S. Q., & Kaunas, R. (2010). Stretch-induced stress fiber remodeling and the activations of JNK and ERK depend on mechanical strain rate, but not FAK. *PloS one*, *5*(8), e12470.

Jungbauer, S., Gao, H., Spatz, J. P., & Kemkemer, R. (2008). Two characteristic regimes in frequency-dependent dynamic reorientation of fibroblasts on cyclically stretched substrates. *Biophysical journal*, *95*(7), 3470-3478.

Chen, K., Hu, X., Blemker, S. S., & Holmes, J. W. (2018). Multiscale computational model of Achilles tendon wound healing: Untangling the effects of repair and loading. *PLOS Computational Biology*, *14*(12), e1006652.

Khayyeri, H., Gustafsson, A., Heuijerjans, A., Matikainen, M. K., Julkunen, P., Eliasson, P., ... & Isaksson, H. (2015). A fibre-reinforced poroviscoelastic model accurately describes the biomechanical behaviour of the rat achilles tendon. PloS one, 10(6), e0126869.

Khayyeri et al., 2017, Achilles tendon compositional and structural properties are altered after unloading by botox. *Scientific reports* 7:13067

Khayyeri, H., Hammerman, M., Turunen, M., Blomgran, P., Notermans, T., Guizar-Sicairos, M., ... & Isaksson, H. (2020). Diminishing effects of mechanical loading over time during rat Achilles tendon healing. bioRxiv.

Turunen et al., 2017, Effects of tissue fixation and dehydration on tendon collagen nanostructure. *Journal of structural biology* 199:209-215

Notermans, T., Khayyeri, H., & Isaksson, H. (2019). Understanding how reduced loading affects Achilles tendon mechanical properties using a fibre-reinforced poro-visco-hyper-elastic model. *Journal of the mechanical behavior of biomedical materials*, *96*, 301-309.

Eliasson, P., Fahlgren, A., Pasternak, B., & Aspenberg, P. (2007). Unloaded rat Achilles tendons continue to grow, but lose viscoelasticity. *Journal of applied physiology*, *103*(2), 459-463.

Richardson, W. J., Kegerreis, B., Thomopoulos, S., & Holmes, J. W. (2018). Potential strain-dependent mechanisms defining matrix alignment in healing tendons. *Biomechanics and modeling in mechanobiology*, *17*(6), 1569-1580.

Wall, M. E., Weinhold, P. S., Siu, T., Brown, T. D., & Banes, A. J. (2007). Comparison of cellular strain with applied substrate strain in vitro. *Journal of biomechanics*, *40*(1), 173-181.

Screen, H. R. C., Lee, D. A., Bader, D. L., & Shelton, J. C. (2003). Development of a technique to determine strains in tendons using the cell nuclei. *Biorheology*, *40*(1, 2, 3), 361-368.

Han, W. M., Heo, S. J., Driscoll, T. P., Delucca, J. F., McLeod, C. M., Smith, L. J., ... & Elliott, D. M. (2016). Microstructural heterogeneity directs micromechanics and mechanobiology in native and engineered fibrocartilage. *Nature materials*, *15*(4), 477-484.

Wilson, W., Van Donkelaar, C. C., Van Rietbergen, B., Ito, K., & Huiskes, R. (2004). Stresses in the local collagen network of articular cartilage: a poroviscoelastic fibril-reinforced finite element study. *Journal of biomechanics*, *37*(3), 357-366.

Tanska, P., Julkunen, P., & Korhonen, R. K. (2018). A computational algorithm to simulate disorganization of collagen network in injured articular cartilage. *Biomechanics and Modeling in Mechanobiology*, *17*(3), 689-699.
